# Current status of invasive mosquito surveillance in the UK

**DOI:** 10.1186/s13071-015-0936-9

**Published:** 2015-06-30

**Authors:** Alexander G. C. Vaux, Jolyon M. Medlock

**Affiliations:** Medical Entomology & Zoonoses Ecology group, Emergency Response Department, Public Health England, Porton Down, Salisbury, SP4 0JG United Kingdom

**Keywords:** *Aedes albopictus*, United Kingdom, Invasive species, Mosquito, Culicidae, Surveillance

## Abstract

**Background:**

Non-native invasive mosquitoes have for many years made incursions into Europe, and are now established in many European countries. The continued European importation of potential vectors and their expansion within Europe increases their potential for importation and establishment in the UK. Coupled with increasing numbers of returning dengue and chikungunya infected travellers, the potential exists for transmission of vector borne disease in new regions.

**Methods:**

To ensure a cost-effective risk assessment and preparedness strategy the UK employs a multi-faceted approach to surveillance for non-native *Aedes* mosquitoes, including passive and active surveillance strategies at a local, regional, and national level. Passive surveillance, including a national mosquito recording scheme and local authority nuisance biting reporting, are combined with targeted active surveillance at seaports, airports, used tyre importers, and motorway service stations.

**Results:**

There is no evidence to date that any invasive *Aedes* species (e.g., *Aedes albopictus*, *Aedes japonicus*, *Aedes aegypti*) occur in the UK despite sharing many of the same routes that have been found to have facilitated their entry into other countries.

**Conclusions:**

This paper sets in context the UK approaches with other European countries and those recommended by the ECDC. It also highlights future UK strategies to enhance surveillance for non-native mosquitoes to help ensure that incursions can be managed, and these mosquitoes do not establish and public health is protected. Focus will be given to increasing the number of submissions of mosquitoes to passive surveillance schemes and maintaining active surveillance efforts at key routes of potential importation.

## Background

In recent decades there has been a re-emergence of vector-borne disease in Europe, including ongoing outbreaks of West Nile virus (WNV) in Southern and Eastern European countries, chikungunya fever in France and Italy, autochthonous transmission of dengue fever in Madeira (Portugal), Croatia and France and vivax malaria in Greece [1, 2]. The increase in trade and transportation of goods and increased movement of humans has dramatically facilitated the importation of both mosquito vector species and mosquito-borne pathogens into Europe. In particular the importation of invasive mosquitoes has been attributed to the global movement of used tyres and wet-footed plants. Coupled with climatic and land-use changes, importation of vectors and pathogens increase the potential for the establishment of non-native and invasive mosquitoes and the consequent vector borne diseases that can result. In 2013 the United Kingdom (UK) reported >550 confirmed cases of travel-related dengue fever and 24 cases of chikungunya fever, and in 2014 > 350 cases of dengue and >300 cases of chikungunya (Public Health England, unpublished data), the latter likely to be related to the outbreak of chikungunya virus (CHIKV) in the Caribbean where 1.4 million cumulative cases have been reported for 2013 and 2014 [3]. Onward transmission of these pathogens is contingent on populations of competent mosquitoes, therefore it is important to establish surveillance and control for these mosquitoes to help ensure that no onward transmission within the UK is possible. In locations in France, where vector-competent invasive non-native mosquitoes are locally established, each imported case of mosquito-borne disease is followed up and if non-native *Aedes* are present then there is comprehensive vector control local to the imported case. This need for mosquito control to limit onward transmission was recently highlighted by local autochthonous transmission of CHIKV [4] and dengue virus (DENV) [5] to French nationals in southern France with no history of travel; with transmission facilitated by recently established *Aedes albopictus* [5].

A number of invasive mosquito species have been introduced into Europe. Their importation to new geographic regions has been on account of their shared life history characteristic of laying drought resistant eggs in human-made containers such as used tyres and wet-footed plants such as lucky bamboo. The global trade in these commodities has facilitated their spread globally. This group of mosquitoes lays eggs that can survive long periods out of water until containers are rewetted at their destination. In this way all the invasive aedine species have been able to colonise new climatically tolerable locations across the globe [6].

**Fig. 1:**
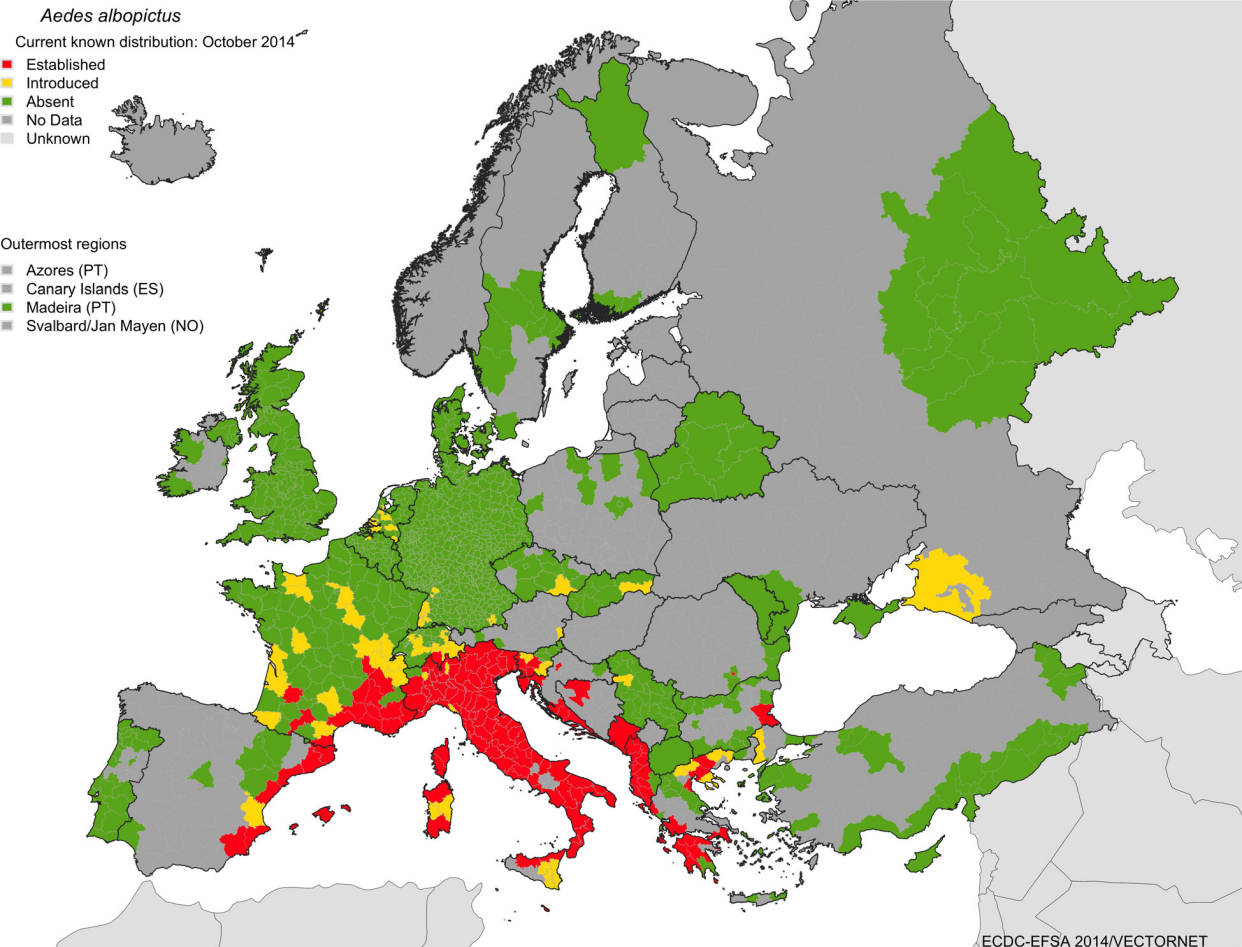
Current known distribution of *Aedes albopictus* as of October 2014. ECDC VBORNET www.ecdc.europa.eu/vbornet

The Asian Tiger mosquito, *Aedes albopictus*, is the most established invasive mosquito species in Europe and has now been reported in 25 European countries including Albania, Austria, Belgium, Bosnia and Herzegovina, Bulgaria, Croatia, Czech Republic, France, Germany, Greece, Italy, Malta, Monaco, Montenegro, the Netherlands, Romania, Russia, San Marino, Serbia, Slovakia, Slovenia, Spain, Switzerland, Turkey and the Vatican City [5]. The species is a particular biting nuisance in many countries including Italy, parts of southern France, Spain, and the Adriatic coasts of Croatia (Fig. 1) [1]. Climate models have shown that the UK’s climate is suitable for the development and sustained maintenance of populations of *Ae. albopictus* [7, 8]. *Aedes albopictus* is a proven vector of CHIKV and has been the primary vector of cases on La Reunion Island in 2005–2007 [9], in Italy in 2007 [10, 11], and in France in 2010 and 2014 [12, 13]. *Aedes albopictus* has also caused outbreaks of DENV on La Reunion Island in 1977–1978 and 2004 [14, 15], Hawaii in 2001–2002 [16], Mauritus in 2009 [17] with the first autochthonous cases in Europe since Greece in 1927 in Croatia in 2010 [18], and France in 2010, 2013 [19, 20], and 2014 [5]. Additional viruses have also been isolated from field specimens of the mosquito, with laboratory transmission demonstrated. These include Eastern equine encephalitis virus (EEEV) [21, 22], La Crosse virus (LACV) [23, 24], Venezuelan equine encephalitis virus (VEEV) [25, 26], West Nile virus (WNV) [27, 28], and Japanese encephalitis virus (JEV) [29].

Five other aedine invasive species have been imported into Europe: *Aedes aegypti*, *Aedes atropalpus*, *Aedes japonicus*, *Aedes koreicus*, and *Aedes triseriatus. Aedes aegypti* was found for many decades in many parts of the Mediterranean, particularly in Italy where it was eradicated in the 1950s [30]. Globally it is the main vector of yellow fever virus (YFV) and DENV, and is also a known vector of CHIKV and Zika virus [1]. The species has recently established on Madeira where it has been implicated in a large outbreak of dengue [30, 31]. *Aedes aegypti* has also been reported in the Netherlands at a used tyre company along with another invasive species *Aedes atropalpus* [32, 33]. *Aedes atropalpus* is native to eastern North America, and has spread in North America via the movement of used tyres [34]. The species is a known vector for WNV [35] and LACV [34], and has been reported and subsequently eradicated in Italy [36], France (S. Chouin & F. Schaffner, unpublished data), and the Netherlands [32]. *Aedes japonicus* was first reported in Europe in France in 2000 where it was eliminated [37, 38], and has since been reported in Belgium [39, 40], and has been found to be established over large areas of Switzerland [38]. It continues to expand its range from Switzerland into Germany along major highway systems, and has been found in cemetery vases where it out-competes local mosquito fauna [41, 42]. It has now been found in Austria, Slovenia [43], Hungary [44], Croatia [5], and Alsace in France [5], showing evidence that the species is continuing to expand its range. *Aedes japonicus* may become a pest species or transmit WNV, and has also shown vector competence for CHIKV and DENV [45]. *Aedes koreicus* has been found in Belgium since 2008 [46] and Italy since 2011 and 2012 [47, 48], and was likely to have been introduced on both occasions as a result of international trade [46]. *Aedes triseriatus* has also been found and controlled at the point of entry in France [1].

Surveillance, defined as a set of procedures implemented in response to a recognised risk [6] is conducted for invasive mosquitoes in European countries by a range of institutions and public bodies, including public health government bodies, environmental health, local and regional councils, food and environment bodies, and private contractors. In the UK, since the issue presents public health concerns at a local and national level, Public Health England (PHE) has played a significant role in surveillance activities. This paper details the approaches taken in the UK, considers them in the context of the ECDC guidance on surveillance for exotic mosquitoes [6], analyses the utility of the various surveillance strategies in the UK and highlights the most appropriate strategies likely to be of most value employed in the UK in the future. The primary aim is to develop economically-viable strategies to help detect, identify and control exotic mosquitoes to prevent their establishment and limit the risk to public health that might ensue.

## Methods

**Fig. 2:**
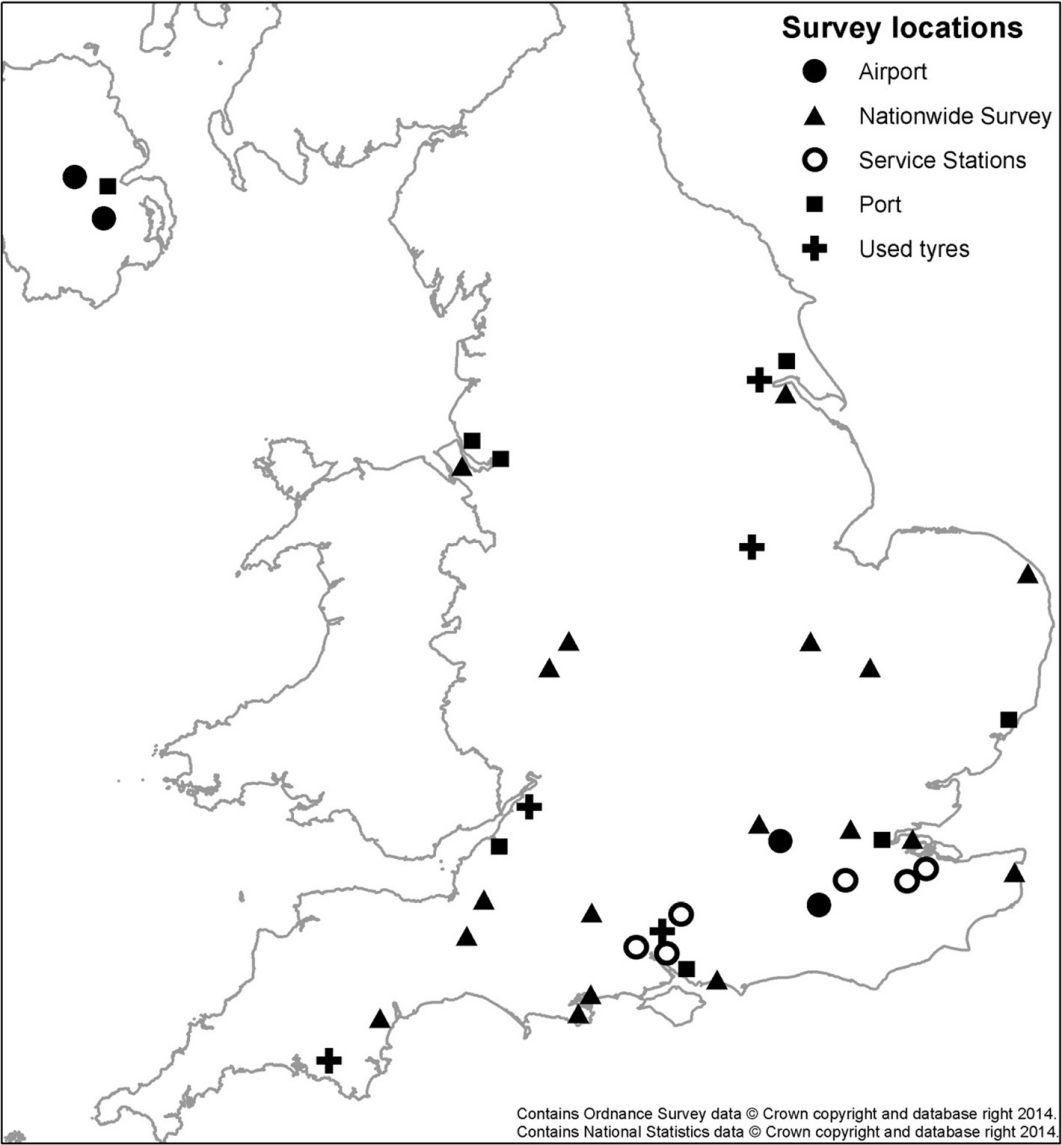
Location of mosquito surveys grouped by Airport, Nationwide Survey, Motorway Service Stations, Port, and Used Tyres

A range of surveillance approaches have been trialled in the UK as part of national efforts to understand the potential risk posed by invasive mosquitoes, and to help in preparedness for detection and control. Surveillance projects have included both passive surveillance (Mosquito Recording Scheme, Mosquito Watch, including the use of a questionnaire based survey) and active surveillance (Nationwide Mosquito Survey, Port Mosquito Surveillance, Used tyre importer surveys, and surveys at motorway service stations) each of which are described separately below. A summary of these strategies is shown in Tables 1 and 2, with a map showing the active surveillance locations in Fig. 2.

**Table 1:** A summary of surveillance strategies employed in the UK

Type of survey	Location	Sampling technique	Lat	Long	County	Frequency	Years
Used Tyres	Ivybridge	BG Sentinel; Ovitraps; larval surveys	50.387	3.954	Devon	Monthly – July to September	2010, 2011, 2012, 2013, 2014
	Grantham	Larval surveys	52.928	−0.655	Lincolnshire	August	2012, 2013
Ports / Airports	Heathrow Airport	Larval surveys	51.471	−0.455	Middlesex	Fortnightly – July to September	2010, 2011, 2012
	Gatwick Airport	Mosquito Magnet; larval surveys	51.154	−0.182	Sussex	Fortnightly – July to September	2010, 2011, 2012
	Felixstowe Port	BG Sentinel; Mosquito Magnet; larval surveys; ovitraps	51.952	1.321	Suffolk	Fortnightly – July to September	2010, 2011, 2012
	Southampton Port	BG Sentinel; Mosquito Magnet; larval surveys; ovitraps	50.903	−1.420	Hampshire	Fortnightly – July to September	2010, 2011
	Manchester Ship Canal	Mosquito Magnet; larval surveys	53.332	−2.756	Greater Manchester	Fortnightly – July to September	2010, 2011, 2012, 2013
	Liverpool Port	Mosquito Magnet; larval surveys	53.456	−3.017	Cheshire	Fortnightly – July to September	2010, 2011, 2012, 2013, 2014
	Belfast City Airport	Larval surveys	54.618	−5.873	Down	August	2010
	Belfast City Port	Mosquito Magnet; larval surveys	54.636	−5.883	Down	Fortnightly – July to September	2010, 2011
	Belfast International Airport	Mosquito Magnet; larval surveys	54.658	−6.216	Antrim	Fortnightly – July to September	2010, 2011
	Bristol Port	Larvae surveys	51.383	−2.719	Avon	July	2010
	Hull Port	Mosquito Magnet	53.741	−0.274	Humberside	Fortnightly – July to September	2010
Motorway Service Stations	Clacket Lane West	Ovitraps	51.271	0.041	Kent	Fortnightly – August to October	2014
	Maidstone	BG Sentinel; Ovitraps	51.266	0.616	Kent	Fortnightly – August to October	2014
	Medway	Ovitraps	51.340	0.609	Kent	Fortnightly – August to October	2014
	Rownhams East	Ovitraps	50.958	−1.447	Hampshire	Fortnightly – August to October	2014
	Rownhams West	BG Sentinel; Ovitraps	50.956	−1.447	Hampshire	Fortnightly – August to October	2014
	Winchester	BG Sentinel; Ovitraps	51.120	−1.254	Hampshire	Fortnightly – August to October	2014
Passive Surveillance	Mosquito Recording Scheme / Mosquito Watch	Submission of records	n/a	n/a	UK-wide	n/a	2005-2014
Survey of Local Authorities	Survey of all 345 Local Authorities	Questionnaire	n/a	n/a	UK-wide	n/a	1970, 1986, 1996, 2009

**Table 2:** Surveillance strategy shown by year

Type of Surveillance	Surveillance Strategy	2009	2010	2011	2012	2013	2014
Passive	Mosquito Recording Scheme	X	X	X	X	X	X
	Mosquito Watch	X	X	X	X	X	X
	Nuisance biting questionnaire	X	X				
Active	Used tyre survey		X	X	X	X	X
	Port mosquito survey		X	X	X	X	
	Nationwide mosquito survey		X	X	X	X	X
	Motorway survey						X

## Results and discussion

### Passive surveillance

#### Mosquito recording scheme

PHE (formerly as one of its now constituent bodies, the Health Protection Agency [HPA]) has run the national Mosquito Recording Scheme (MRS) since 2005, contributing to species recording efforts in collaboration with the Biological Records Centre (BRC). The MRS aims to create a forum for storing data on mosquito distribution including historical datasets as well as more recent data contributed by amateur and professional entomologists, museums, universities, the public and government entomologists. The recording scheme, including its predecessor run by the British Mosquito Group, has gathered >10,000 records of all 34 British mosquito species, with data comprising ~3500 submissions, and an additional 7000 records from historical datasets with records as far back as the 1850s (Table 3) [49–56]. The MRS data is made publically accessible via the NBN Gateway (https://data.nbn.org.uk). Counties with the highest numbers of species are those in the south-east of England, with Dorset, Hampshire, London, Kent, and Essex all having records of more than 21 species of mosquito [49]. This scheme provides a forum for submitting records and samples of British mosquitoes, but so far no invasive species have been submitted. The scheme has generated a wealth of data, however as a passive method it has its limitations, chiefly that the number of records submitted for particular locations can reflect both survey effort as well as mosquito abundance. However it does provide a method for people to have mosquitoes identified by medical entomologists, and importantly for PHE, provides a way to identify nuisance and/or invasive species. It also establishes a database upon which national risk assessments can be informed and changes in species distribution monitored. This approach drastically increases the potential geographical range of sampling, over that which would be cost effective through more labour intensive approaches.

**Table 3:** Records submitted to the Mosquito Recording Scheme

Species	Number of records
*Aedes vexans*	36
*Anopheles algeriensis*	173
*Anopheles atroparvus*	111
*Anopheles claviger*	779
*Anopheles daciae*	472
*Anopheles maculipennis s.l*.	1220
*Anopheles messeae*	127
*Anopheles plumbeus*	259
*Coquillettidia richiardii*	310
*Culex modestus*	13
*Culex pipiens s.l*.	1371
*Culex pipiens molestus*	96
*Culex territans*	43
*Culex torrentium*	429
*Culiseta annulata*	834
*Culiseta fumipennis*	70
*Culiseta litorea*	46
*Culiseta longiareolata*	44
*Culiseta morsitans*	494
*Culiseta subochrea*	57
*Dahliana geniculata*	330
*Ochlerotatus annulipes*	214
*Ochlerotatus cantans*	661
*Ochlerotatus caspius*	219
*Ochlerotatus communis*	20
*Ochlerotatus detritus*	476
*Ochlerotatus dorsalis*	56
*Ochlerotatus flavescens*	37
*Ochlerotatus leucomelas*	4
*Ochlerotatus punctor*	556
*Ochlerotatus rusticus*	245
*Ochlerotatus sticticus*	8
*Orthopodomyia pulcripalpis*	52
Total	10,099

PHE is actively working to develop and promote this scheme further, to encourage the submission of records and reports of nuisance biting. This will be key to improving the understanding of British mosquito species causing a biting nuisance, and will further contribute to preparedness for potential incursion of mosquito borne pathogens that might be vectored by British mosquitoes. The MRS may also provide the mechanism for the identification of invasive species, since a similar scheme provided the first indication of the presence of *Aedes japonicus* in Germany [38].

#### Mosquito watch

In a similar vein to the MRS, Mosquito Watch was a scheme set up by the HPA in 2005, in collaboration with the Chartered Institute of Environmental Health (CIEH), and Killgerm Ltd, to better understand the incidence of biting mosquitoes in the UK by encouraging Environmental Health Officers (EHOs) to record nuisance mosquitoes and submit samples for identification. Prior to this scheme there was no mechanism that allowed EHOs to have their samples identified nor for the records to be collated. One of the aims of Mosquito Watch was to provide an early detection system for invasive species that might be causing a nuisance. Since 2010 Mosquito Watch data has been added to and merged with the MRS data. The scheme provides the medical entomology resource required when EHOs are investigating a local mosquito nuisance biting issue and ensures PHE is up-to-date regarding local mosquito biting issues. Between 2005–2010, there were 116 mosquito reports to the scheme, dominated by *Culiseta annulata* (56), and *Culex pipiens* s.l. (42), with some records of *Ochlerotatus detritus* (7), non-specific *Aedes*/*Ochlerotatus* spp. (7), *Coquillettidia richiardii* (1) and *Anopheles maculipennis* s.l (1) [57]. Despite several initial false alarms, there were no confirmed reports of invasive *Aedes* species once specimens had been reliably identified. *Culiseta annulata* is known to be a biting nuisance in the UK, and while *Culex pipiens* biotype *molestus* is also a nuisance species, most of the records of the ornithophilic *Culex pipiens* s.l. have been submitted in late September when the typical form of this mosquito enters people’s dwellings for hibernation. On several occasions, the public have been concerned about the potential presence of *Ae. albopictus*, which often generated press interest. However following rapid identification through Mosquito Watch they were in every case identified as *Cs. annulata*. It is likely that in the event of an invasive species causing nuisance that the public and EHOs will be first to be alerted to it.

#### Nuisance biting survey

In order to better understand the rate of nuisance biting and the incidence of hitherto unreported localities of nuisance biting mosquitoes, the HPA in collaboration with the University of East London, and CIEH, conducted a questionnaire survey in 2009 across all UK local authority (LA) environmental health departments responsible for mosquito control [57]. This questionnaire asked LA pest control officers various questions on the reported incidence of nuisance mosquito biting, the mosquito species implicated, whether aquatic breeding sites were identified, and whether mosquito control strategies were employed. Two-hundred and twenty-one LAs (64 % return) completed the questionnaire; 57 LAs reported evidence of mosquito nuisance incidence in the last 10 years, and 29 during the last 12 months. There was no evidence of nuisance biting as a result of invasive species, however when compared with similar surveys conducted in the 1980s and 1990s [57], data from 2009 showed a 2.5-fold increase in reports to LAs over the last 10 years of nuisance biting by native mosquitoes. The most common nuisance species with confirmed identification were *Culiseta annulata*, *Ochlerotatus detritus*, *Culex pipiens* s.l., *Ochlerotatus cantans* and *Anopheles maculipennis* s.l.. Mosquito control had been conducted by 11 LAs including sites at water treatment and sewage works, coastal wetlands and saltmarshes. All of these LAs used larvicidal control methods, with three specifically reporting use of microbial larvicide *Bacillus thuringiensis israeliensis* (Bti) toxin. Other strategies included habitat management, netting, trapping, flushing of drains, and decommissioning filter beds.

### Active surveillance

#### Port mosquito surveillance

**Fig. 3:**
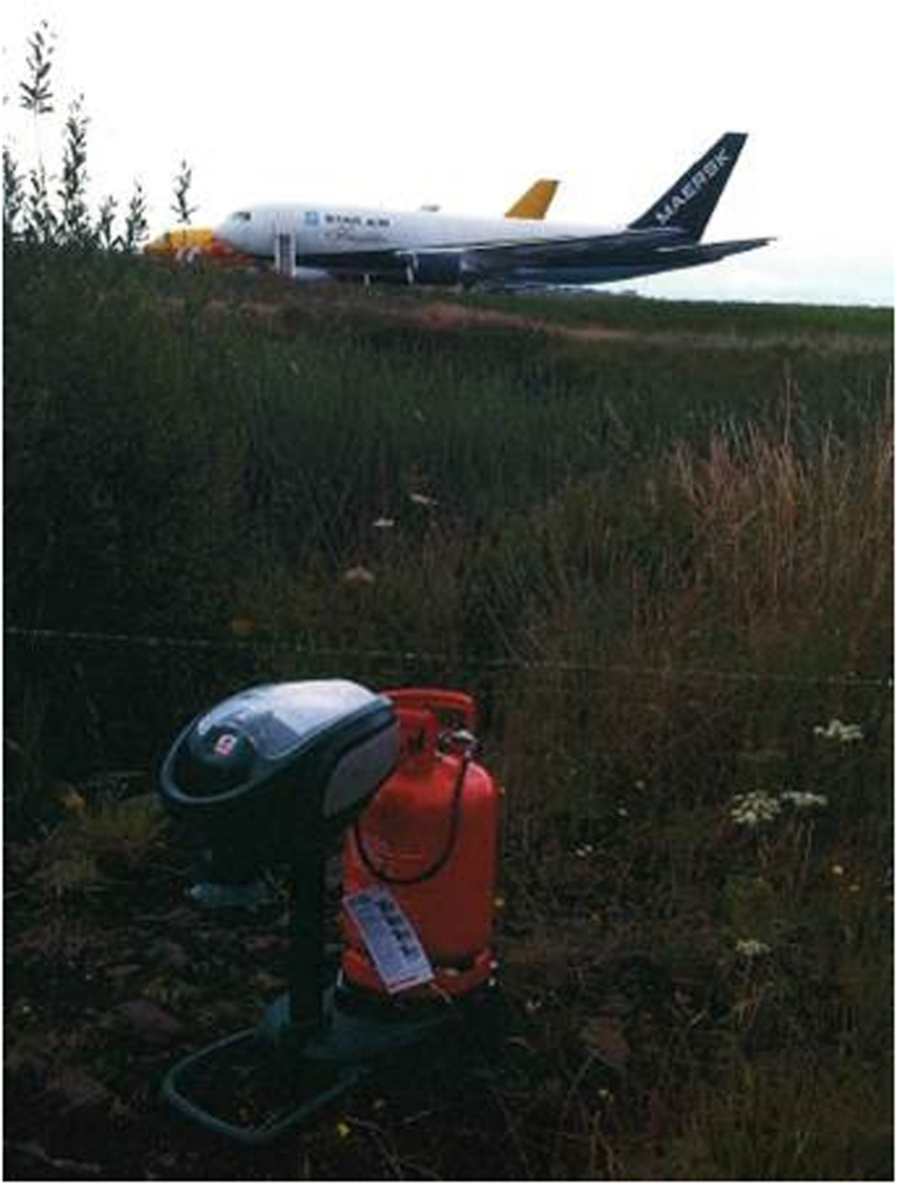
Photograph of Mosquito Magnet at an airport

**Fig. 4:**
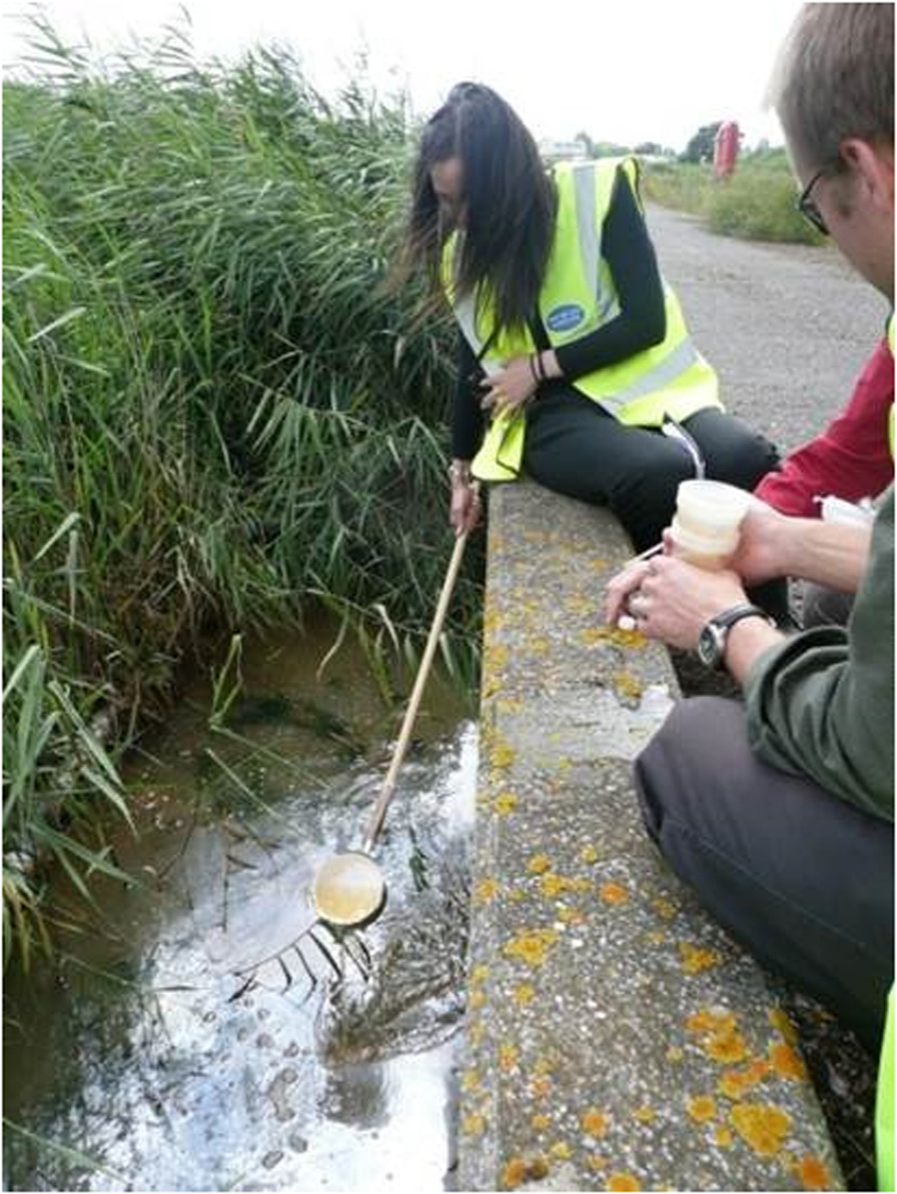
Photograph of larval surveys at London Heathrow airport

A joint pilot initiative was set up between PHE, Salford University, the Association of Port Health Authorities (APHA), and eleven sea and airports throughout England and Northern Ireland, to examine the potential for imported mosquitoes. Published in two papers [58, 59], the aim of this project was to investigate the level of preparedness of UK seaports and airports for exotic invasive mosquitoes and assess the likelihood of sea and airports as routes of invasive mosquito importation. The objectives of the study were (1) to establish a baseline understanding of the aquatic mosquito habitats in and around major ports in England and Northern Ireland, (2) to conduct active surveillance for invasive mosquitoes at the ports, (3) to identify appropriate surveillance methodologies suited to port environments, and (4) to develop the capability and capacity of Port Health Officers (PHOs) to conduct invasive mosquito surveillance. Surveys were conducted at London Heathrow airport, London Gatwick airport, Belfast International and Belfast City airports, Felixstowe seaport, Southampton seaport, Bristol seaport, Hull seaport, Manchester seaport, and Liverpool seaport (Fig. 2, Tables 1 and 2). PHOs mapped and surveyed a wide range of different potential aquatic habitats and container habitats within their ports for mosquitoes. Ovitraps were used at the ports where possible, BG Sentinel adult traps (Biogents, Regensburg, Germany, http://www.biogents.com/) baited with Sweetscent® lures (Biogents, Germany), and the Mosquito Magnet® Executive Mosquito trap (MosquitoMagnet, Lititz, Pennsylvania, USA; http://www.mosquitomagnet.com/) (Fig. 3) were also used where appropriate and possible. The choice of sampling methods (larval surveys (Fig. 4), ovitraps, Mosquito Magnet, BG Sentinel) was driven by particular characteristics of each seaport / airport as permitted by their security policy and accessibility, and also the human resources available [59].

**Fig. 5:**
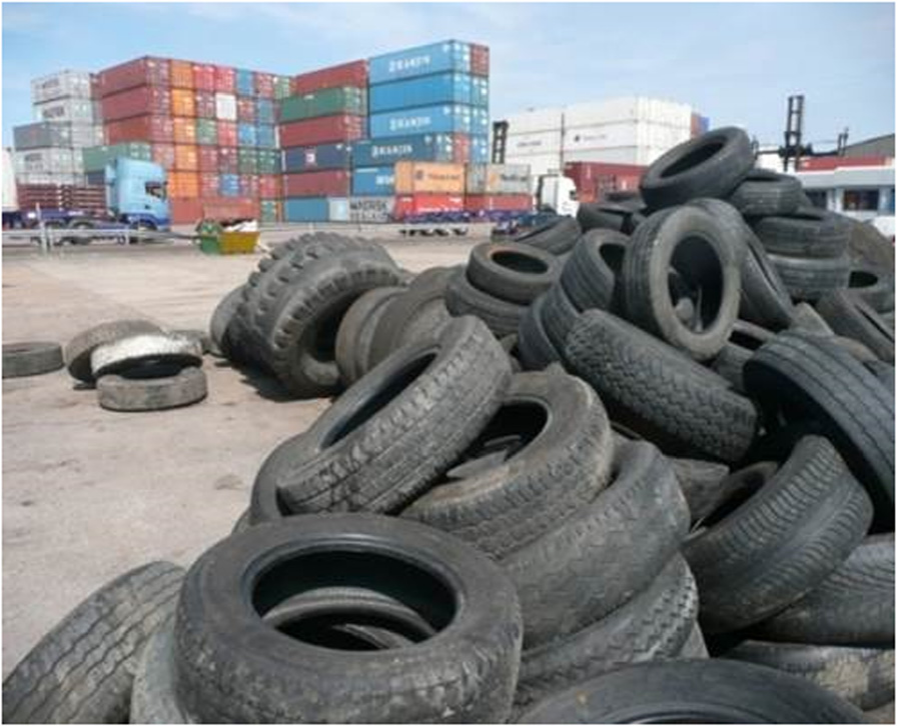
Photograph of used tyres at a seaport

The port mosquito survey was started in 2010 and where possible mosquito surveillance continued over the subsequent years. No invasive mosquitoes have so far been recorded, and of the six native species that were recorded during the intensive survey in 2010, all are widespread and abundant throughout the majority of the UK (*Culex pipiens* s.l., *Anopheles claviger*, *Culiseta annulata*, *Anopheles maculipennis* s.l., *Ochlerotatus detritus*, and *Coquilletidia richiardii*). The main aim of the project was to facilitate each PHO to identify the optimal surveillance technique for their seaport / airport, particularly regarding operationally intensive areas surrounding aircraft and container movements (Fig. 5). BG Sentinel traps provided a useful and efficient method of monitoring adult mosquitoes in sheltered areas such as imported goods warehouses, with ovitraps used in key locations around cargo areas. Mosquito Magnets continue to be used at Liverpool seaport, and BG Sentinels have also had further use at Felixstowe Border Inspection Post (BIP) where food and other produce imports are inspected. The project identified a number of key areas where further efforts are required including: a focus on the importation of used tyres and development of a database that tracks the movement of these goods; continued reporting of nuisance biting to the Mosquito Recording Scheme; and capacity building to apply control regimes in the event of finding an invasive species.

PHE has also trialled additional surveillance at three of the UK’s largest seaports at Felixstowe, Southampton, and Tilbury (London) (Fig. 2). As described in the ECDC Guidelines for the Surveillance of Invasive Mosquitoes [6], ovitraps are recommended for use in a variety of survey methodologies for invasive *Aedes* species, particularly in relation to seaports and airports. Twenty locations were identified in the vicinity of each seaport, with an ovitrap placed in each location. Each ovitrap was half-filled with water and a floating polystyrene block to facilitate ovipositing female *Aedes* mosquitoes to lay eggs. Traps were located during early September 2011 in vegetated areas close to the seaports’ boundaries and along the major access routes and collected during the first week of October 2011 after four weeks deployment. Locations were chosen based on their proximity to the port boundaries, using aerial photographs and port boundary maps, and within areas of vegetation where adult invasive mosquitoes might rest. None of the 60 ovitraps had any mosquito eggs or larvae. This type of surveillance ideally requires a regular commitment of resources to enable fortnightly checks of ovitraps from July to October, at an approximate density of 1/5000 m^2^, targeting points of entry and storage sites for imported goods relevant to invasive mosquitoes such as used tyres or lucky bamboo [6]. Finding suitable discreet locations for siting ovitraps around ports, particularly where there is a lot of activity, can be problematic. Furthermore, given that seaports are rarely the final destination for relevant imported goods such as used tyres, it was considered more efficient and effective to focus efforts on surveillance around used tyre imports. Ovitrapping around seaports was not continued after 2011.

#### Nationwide mosquito surveillance

As part of their ongoing vector surveillance activities PHE monitor native mosquitoes through a network of Mosquito Magnet traps run at nature reserves across England (Fig. 2; Tables 1 and 2). Although this project does not target exotic invasive mosquitoes specifically it aims to provide seasonality and abundance data on a range of native mosquito species, particularly those that have been identified as being of public health significance in other countries. In the four years of the project, more than 15,000 mosquitoes have been collected. At one of the sites, *Culex modestus* was trapped in considerable numbers [60]. This species was not previously considered native to the UK, but has now been found in further surveys in Cambridgeshire, Dorset, and Essex [61–63], and as a key vector for WNV in Europe, the presence of this species is significant in terms of WNV risk assessment. These findings highlight the importance of running a trapping network to ensure the data on presence and status of key mosquito species is accurate and updated. Indeed using similar routine mosquito surveillance in this way, *Aedes japonicus* has been recorded in the Netherlands [64], and *Aedes koreicus* and *Aedes japonicus* in Belgium [65].

#### Used tyre mosquito surveillance

**Fig. 6:**
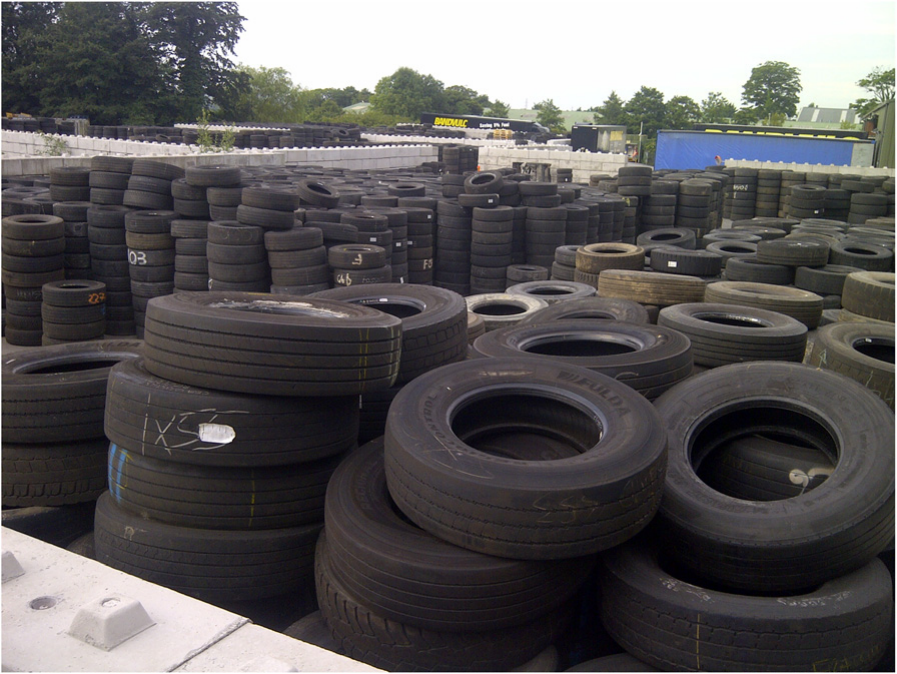
Photograph of used tyres stored at a retreaded tyre manufacturer

Surveillance for invasive mosquitoes has also been conducted by PHE at several major used tyre importers annually since 2010 (Tables 1 and 2; Fig. 6). An initial search of imported tyre companies was conducted by systematically identifying tyre importers via internet and telephone book searches, existing databases and reports. HM Customs data was available for the years 2009–2012 for all companies importing used tyres from outside the EU, and letters were sent to all to arrange access for surveys. Although the return rate was low, surveys were conducted at five companies including the two largest importers of used tyres (including truck tyres for retreading), and regular surveys have been made each summer since (Fig. 6). During June to September used tyres stored at the yards of the two largest importers are sampled for mosquito larvae, with additional ovitraps and BG-Mosquitaire adult traps (Biogents, Regensburg, Germany, http://www.biogents.com/) placed in line with ECDC Guidelines [6]. To date no invasive species have been found during these surveys. *Culex pipiens* s.l. is most commonly found, and *Culiseta annulata* is frequently found in tyres with high amounts of leaf litter and vegetation. Mosquito larvae are most often found closest to the perimeter of the tyre storage yard, which may be a result of proximity to vegetation, or because the tyres have in some cases been stored there the longest.

The majority of companies that import used tyres do so for the specific purpose of retreading which is undertaken relatively swiftly, which means that most of the tyres do not sit in the yard for long and therefore there is minimal time for pre-imaginal development. This may differ from sites elsewhere, such as those in the Netherlands where invasive exotic mosquitoes have been found in tyres that have been left outside for a number of months (E-J. Scholte, *Per comm*.). Surveillance at the main imported tyre companies in England will continue, as these provide sentinels for any change in the importation of tyres. There remains an issue however over the exact destinations of all imported tyres, and until this data is accessible and reliable it will not be possible to conduct such thorough surveys as those currently conducted in the Netherlands.

#### Motorway service stations

More recently there has been a shift in the means of dissemination of *Aedes albopictus* to new geographic areas in Europe (i.e. northward movement along highways in France) so that the species is just as likely to be imported into the UK from continental Europe through road networks (i.e. ferry ports / Eurotunnel) as through ports on tyres. Passive movement of *Aedes albopictus* through vehicular movements has been identified as the main route by which adult mosquitoes have been moved to new regions, and is likely to have been the case in Austria, the Balkans, the Czech Republic, southern France, Germany, Spain and Switzerland [66–70]. *Aedes albopictus* has been recorded in new regions at motorway service stations, where, it is suggested that holiday makers in their cars and caravans, as well as other road users, move adult mosquitoes across borders within their vehicles, and at rest stops and service areas the mosquitoes leave the vehicles and fly to nearby habitat. In recent years, the northward distribution of *Aedes albopictus* in France has raised the prospect of vehicles as a prime route for importing these mosquitoes through UK ferry ports.

**Fig. 7:**
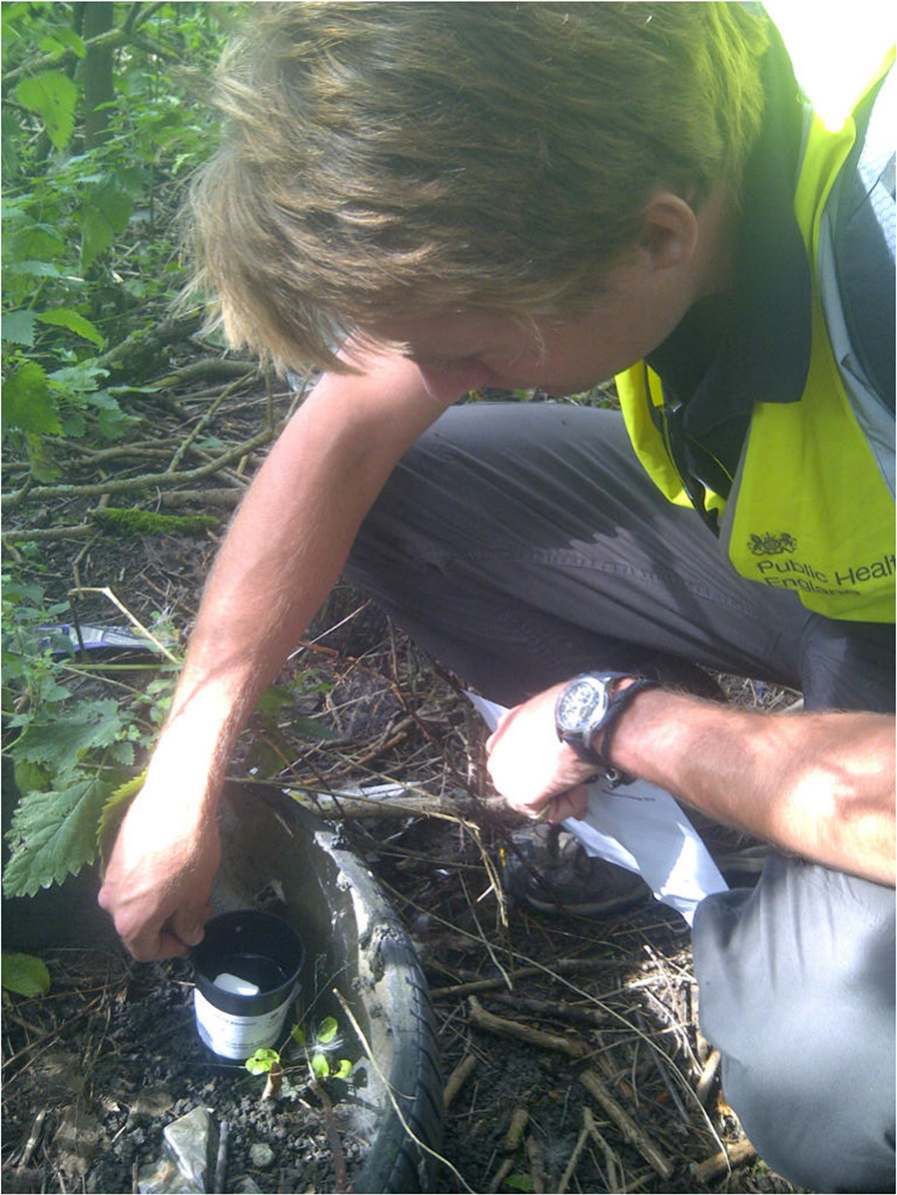
Photograph of an ovitrap at a motorway service station. All photographs attributed to Medical Entomology and Zoonoses Ecology group (MEZE); Public Health England

UK ferry ports are not considered to be appropriate for mosquito surveillance, given that vehicles exiting ferries or Eurotunnel are not required to stop for any significant period of time. Therefore focus has been given to motorway service stations along the inland routes from the ferry ports in southern England and Eurotunnel terminals. Invasive mosquito surveillance was therefore initiated by PHE at six service stations in the UK from August to October 2014 (Fig. 2; Tables 1 and 2). They were all chosen based on their proximity to cross-channel connections, and because they served traffic having recently arrived through the Eurotunnel and cross-channel ferry ports. A total of 56 ovitraps (Fig. 7) were set out across the six service stations from August to October, in vegetation around the car, caravan, and lorry parking areas. At three services stations, BG Sentinel adult traps were deployed using Sweetscent® lures. The ovitraps and adult traps were checked every two weeks until the second week of October. The substrate used in the ovitraps, cubes of polystyrene (~5 cm^3^), were visually checked for eggs, and if warranted, were placed in a labelled sample bag and returned to the laboratory for further examination under the microscope. Adult trap catch bags were replaced every two weeks and the catch examined and sorted in the laboratory. No mosquito eggs were found, and the only adult species found in the adult traps was the native and ubiquitous *Culex pipiens* s.l..

## Conclusion and recommendations

In order to detect invasive mosquitoes, the UK has employed a multi-faceted approach to surveillance adopting both passive and active surveillance methods. ECDC has recently published guidelines for the surveillance of invasive mosquitoes in Europe [6] and it is in this context that the UK’s approach is considered in this paper. The ECDC guidelines make recommendations for surveillance methods depending on the aims of the surveillance programme and the status of invasive species and mosquito borne diseases in the country.

The UK position regarding surveillance has not previously been defined in terms of the broad actions as set out in the ECDC guidelines, however many of the functions are currently conducted by a range of organisations and government bodies. In general terms, given the risk that invasive exotic mosquitoes present to the UK public health, the main cause for concern lies with public health, and so far much of the surveillance effort has been conducted by the PHE medical entomology group. The recent outbreak of chikungunya virus in the Caribbean, and the ongoing spread of invasive mosquitoes and incidence of autochthonous arbovirus transmission in Europe highlights the public health concerns associated with invasive mosquitoes. Given the broad range of stakeholders across both industry and government, an inclusive strategy is now being developed to prepare for and respond to the risk that exotic invasive mosquitoes may pose to the UK.

The World Health Organization publication *The Regional Framework for surveillance and control of invasive mosquito vectors and re*-*emerging vector*-*borne diseases 2014*–*2020* [71] also highlights the importance of prevention, surveillance, and control of invasive mosquito species, and raises the need for harmonisation of methods and procedures for prevention, surveillance, and control across the European region. For the UK, given that there have been no reports of invasive mosquitoes, the recommendations are for an active surveillance programme to detect possible introductions at Points of Entry (PoE) such as used tyre importers, airports, seaports, and major ground transportation routes. In addition to surveillance at used tyre importers, and owing to a change in distribution, it is warranted that focus should now also be given to vehicular movement of mosquitoes via ground transportation routes.

The ECDC Guidelines also recommend the development of active surveillance in areas reporting mosquito-borne disease and that information procedures and mosquito elimination plans should be prepared for the event that invasive mosquitoes are found. In the UK there have so far been no known cases of autochthonous dengue or chikungunya fever, however the development of procedures and protocols to manage and eradicate invasive mosquitoes are the subject of ongoing discussions between PHE and stakeholders. Given the large number of imported cases of DENV and CHIKV, maintaining the absence of invasive mosquitoes is paramount. In the event that invasive mosquitoes are detected in the UK and found to be established, a plan would then need to be enacted to control mosquitoes around imported cases. This is the current strategy in France, and although the climate may be more permissible in southern France for onward arboviral transmission and for increased vector densities, the potential for onward transmission in the UK would need to be considered in the event that invasive mosquitoes became established and constituted a nuisance risk.

Invasive mosquito surveillance has identified the need to maintain and enhance the existing methods of surveillance used in the UK to ensure incursions of exotic mosquitoes are identified at an early stage of their introduction, thereby allowing control measures to be successful. Mosquito surveillance at seaports and airports has shown how these vast areas of varied activity can support a range of mosquito species, and crucially have shown that efforts should continue to be targeted on relevant goods imports. The identification of relevant imported goods to mosquito surveillance is therefore required at an early stage, to enable particular importers (e.g., of used tyres) to be part of an ongoing surveillance project.

Future plans for surveillance of invasive mosquitoes now centre on:

- Developing a database of importers of relevant goods, and regular surveillance of importers.
- Continued monitoring of UK ports and airports, using traps targeted at imported goods
- Continued surveillance of motorway service stations to detect mosquitoes entering through ferry ports and the channel tunnel.
- Continuation and development of the mosquito recording scheme and mosquito watch to ensure cases of nuisance biting are reported.
- Engagement of all stakeholders towards the development of a national invasive mosquito control strategy that ensures the UK is ready to rapidly respond to the findings of invasive species with a robust and effective control strategy.

The development of surveillance strategies and preparedness in line with the relevant guidelines and experiences in the European region for identifying and responding to exotic invasive mosquitoes in the UK is a multi-stakeholder task and requires a coherent and planned approach, including engagement with the industry. Additional surveillance work and development of UK centric plans is an ongoing commitment to UK public health and with proportionate preparedness the risk of establishment of exotic invasive mosquitoes will be minimised.
